# Supplementary material for: TCAF1 promotes TRPV2-mediated Ca2+ release in response to cytosolic DNA to protect stressed replication forks
Source: Nat Commun. 2024 May 30;15:4609. doi: 10.1038/s41467-024-48988-6 (PMC11139906; doi:10.1038/s41467-024-48988-6)
Supplement: Supplementary file 1 — Supplementary Information [file 41467_2024_48988_MOESM1_ESM.pdf]

Supplementary Information

TCAF1 Promotes TRPV2-mediated Ca^{2+} Release in Response to Cytosolic DNA to Protect Stressed Replication Forks

Lingzhen Kong[1*], Chen Cheng[1*], Abigael Cheruiyot[1], Jiayi Yuan[1], Yichan Yang[1], Sydney Hwang[1], Daniel Foust[1], Ning Tsao[2], Emily Wilkerson[1], Nima Mosammaparast[2], M. Ben Major[1], David W. Piston[1] and Shan Li[1, 3,4#], Zhongsheng You[1#]

Contents

Supplementary Figures

Supplementary Figure 1 2
Supplementary Figure 2 4
Supplementary Figure 3 6
Supplementary Figure 4 7

Supplementary Figure 1 (related to Figure 1)

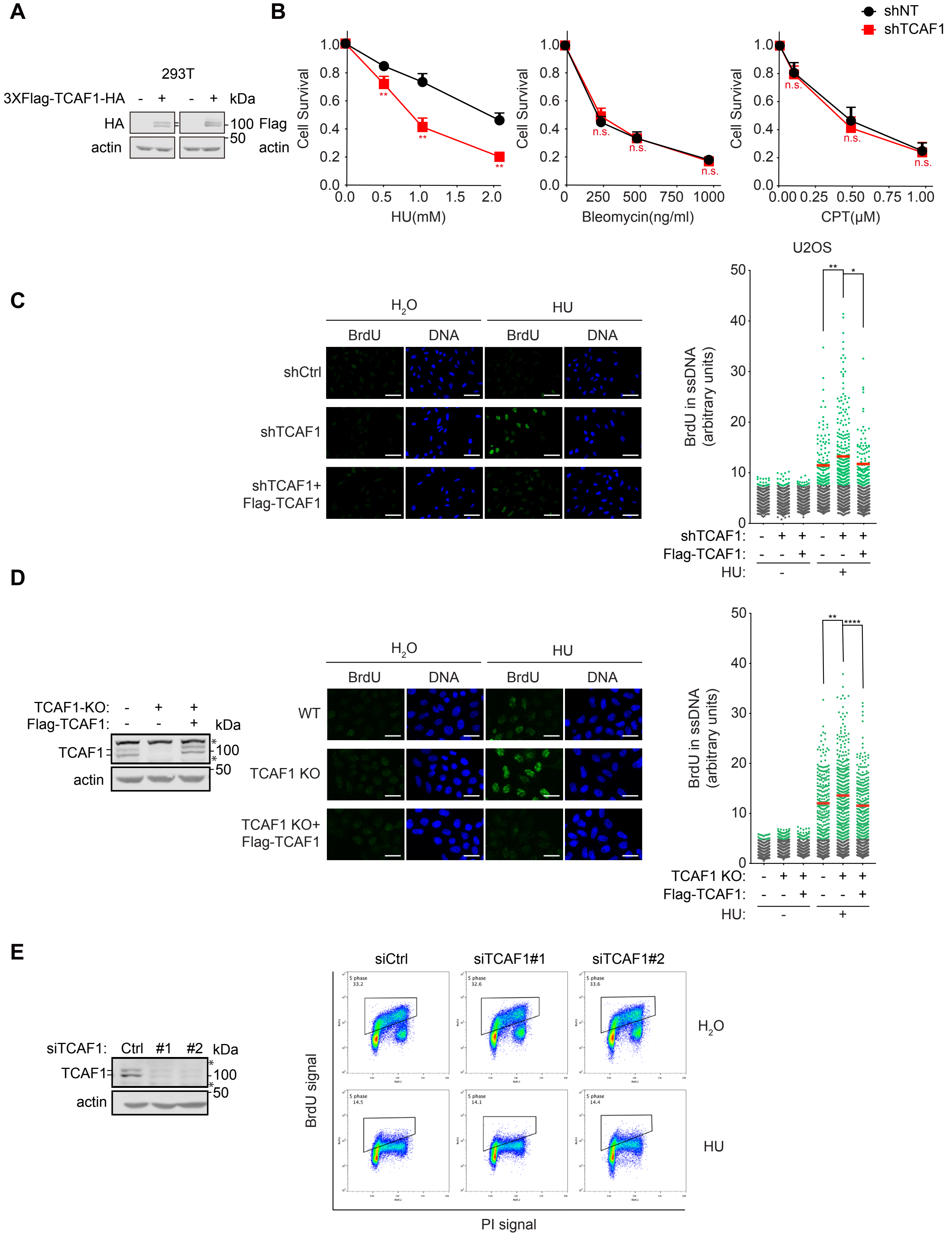

Supplementary Fig.1. TCAF1 protects replication forks after replication stress (Related to Figure 1).

(A) Western blot analysis showing the western blot band pattern of N-terminally 3×Flag tagged and C-terminally HA-tagged TCAF1 expressed in 293T cells.

(B) Effects of TCAF1 knockdown on cell survival after treatment with indicated concentrations of HU (24 h), bleomycin (24h), or CPT (4 h). Data represent mean ± S.D. from triplicates. **, p≤0.01, n.s., not significant (two-tailed, unpaired t-test).

(C) Effects of TCAF1 knockdown on fork resection in U2OS cells and the rescue of fork resection by Flag-TCAF1 expression after HU treatment (2 mM, 5 h). Left panel: Representative native BrdU IF images for fork resection (scale bar, 25 μm). Right panel: Quantified BrdU signal in ssDNA in the samples depicted in the left panel. Cells with a BrdU signal higher than the majority (98%) of H_2O-treated control cells (gray dots) were taken as BrdU-positive (green dots). Red bars represent the mean BrdU intensity of BrdU-positive cells. At least 1,000 cells were analyzed for each sample. n=3, **, p≤0.01. *, p≤0.05 (two-tailed, unpaired t-test).

(D) Left panel: Western blot analysis of CRISPR/Cas9-mediated TCAF1 knockout and the expression of Flag-TCAF1 in TCAF1-KO HeLa cells. *, nonspecific bands. Middle panel: Representative BrdU IF images of cells treated with HU (2 mM, 5 h) or H_2O (scale bar, 25 μm). Right panel: Quantified BrdU signal in ssDNA in the samples depicted in the middle panel. Cells with a BrdU signal higher than the majority (98%) of H_2O-treated control cells (gray dots) were taken as BrdU-positive (green dots). Red bars represent the mean BrdU intensity of BrdU-positive cells. At least 1,000 cells were analyzed for each sample. n=3, ****, p≤0.0001. **, p≤0.01 (two-tailed, unpaired t-test). Outliers were removed through ROUT (Q=1%) analysis.

(E) Effects of TCAF1 knockdown on the cell cycle in HeLa cells. Left panel: Western blot analysis of TCAF1 knockdown by two different siRNAs in HeLa cells. Right panel: HU-treated (4 mM, 4 h) or -untreated cells were pulse-labeled with BrdU for 30 min. Flow cytometry analysis was performed after BrdU IF under a denaturing condition and propidium iodide (PI) staining.

Source data are provided as a Source Data file.

Supplementary Figure 2 (related to Figure 2)

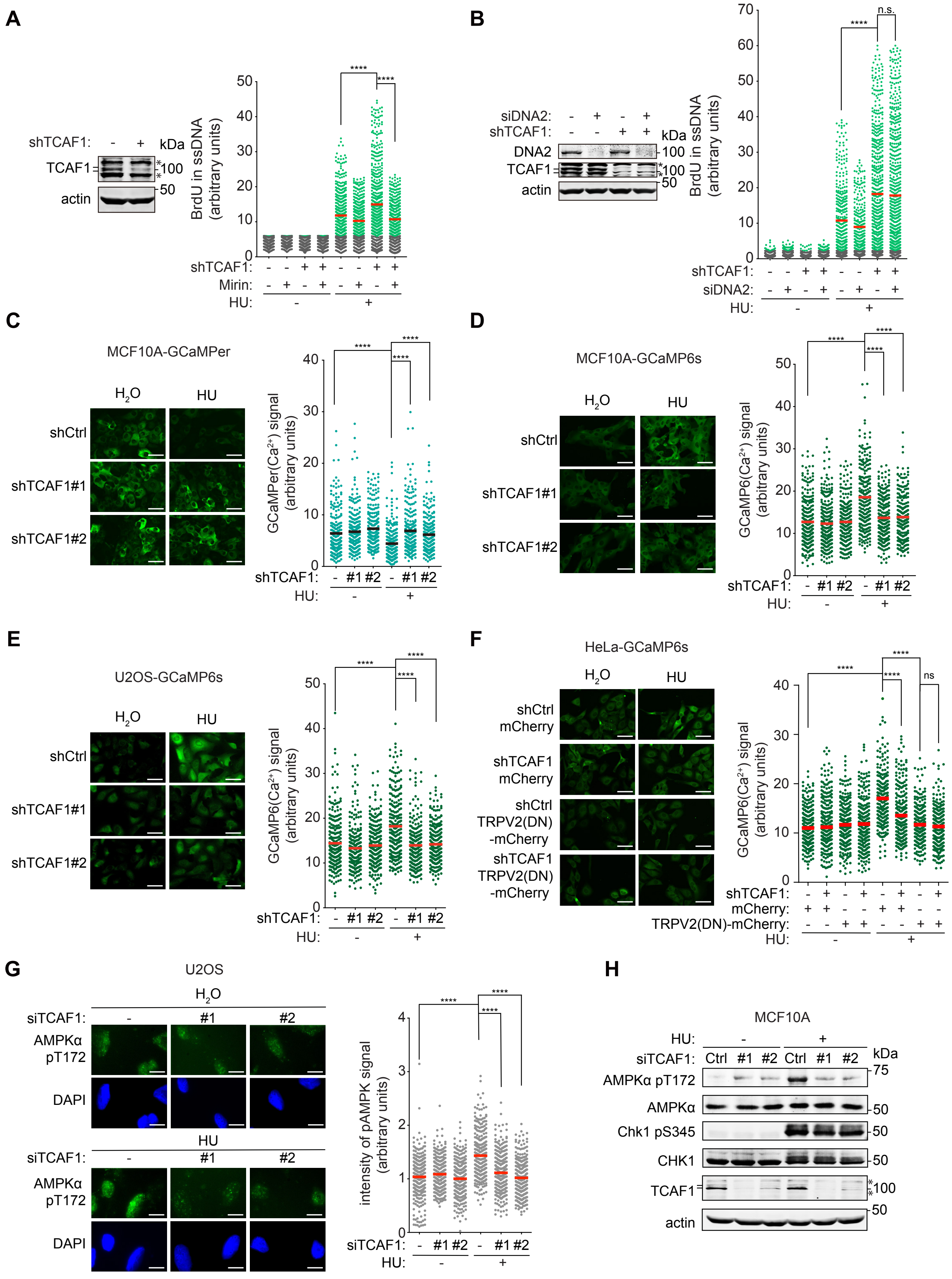

Supplementary Fig.2. TCAF1 protects replication forks through the Ca^{2+}-CaMKK2-AMPK-Exo1 signaling pathway (Related to Figure 2).

(A) Effects of Mre11 inhibition on fork resection in control-knockdown and TCAF1-knockdown HeLa cells. Left panel: shRNA-mediated knockdown of TCAF1 in HeLa cells. *, nonspecific bands. Right panel: quantified BrdU signals in control-knockdown and TCAF1-knockdown HeLa cells treated with HU (2 mM) together with Mirin (50 mM) or DMSO for 5 h. Red bars represent the mean BrdU intensity of BrdU-positive cells. At least 1,000 cells were analyzed for each sample. n=3, ****, $p \leq 0.0001$ (two-tailed, unpaired t-test).

(B) Effects of DNA2 knockdown on fork resection in control-knockdown and TCAF1-knockdown HeLa cells. Left panel: siRNA-mediated knockdown of DNA2 in control-knockdown and TCAF1-knockdown HeLa cells. *, nonspecific bands. Right panel: quantified BrdU signals in cells treated with HU (2 mM, 5 h) or H_2O. Red bars represent the mean BrdU intensity of BrdU-positive cells. At least 1,000 cells were analyzed for each sample. n=3, ****, $p \leq 0.0001$. n.s., not significant (two-tailed, unpaired t-test).

(C) Effects of TCAF1 knockdown on HU-induced ER Ca^{2+} release in MCF10A cells. Left panel: Representative images of the GCaMPer signal (scale bar, 25 μm). Right panel: Quantified GCaMPer signal in S phase-synchronized cells after HU treatment (4 mM, 4 h). 250 cells were scored for each sample. Black bars represent the mean. n=3, ****, $p \leq 0.0001$ (two-tailed, unpaired t-test).

(D) Effects of TCAF1 knockdown on HU-induced iCa^{2+} elevation in MCF10A cells. Left panel: Representative images of the GCaMP6s signal (scale bar, 25 μm). Right panel: Quantified GCaMP6s signal in S phase-synchronized cells after HU treatment (4 mM, 4 h). 250 cells were scored for each sample. Red bars represent the mean. n=3, ****, $p \leq 0.0001$ (two-tailed, unpaired t-test).

(E) Effects of TCAF1 knockdown on HU-induced iCa^{2+} elevation in U2OS cells. Left panel: Representative images of the GCaMP6s signal (scale bar, 25 μm). Right panel: Quantified GCaMP6s signal in S phase-synchronized cells after HU treatment (4 mM, 4 h). 250 cells were scored for each sample. Red bars represent the mean. n=3, ****, $p \leq 0.0001$ (two-tailed, unpaired t-test).

(F) Effects of TCAF1 knockdown on HU-induced iCa^{2+} elevation in HeLa cells expressing mCherry or TRPV2(DN)-mCherry. Left panel: Representative images of the GCaMP6s signal (scale bar, 25 μm). Right panel: Quantified GCaMP6s signal in S phase-synchronized cells after HU treatment (4 mM, 4 h). 250 cells were scored for each sample. Red bars represent the mean. n=3, ****, $p \leq 0.0001$ (two-tailed, unpaired t-test).

(G) Effects of TCAF1 knockdown on HU-induced T172-phosphorylation of AMPKα in U2OS cells. Left panel: Representative images of T172-phosphorylation of AMPKα immunofluorescence signal after treatment with HU (4 mM, 4 h) or H_2O (scale bar, 10 μm). Right panel: Quantified T172-phosphorylation of AMPKα immunofluorescence signal in cells treated with HU (4 mM) or H_2O for 4 h. At least 250 cells were scored for each sample. Red bars represent the mean. n=3, ****, $p \leq 0.0001$ (two-tailed, unpaired t-test).

(H) Effects of TCAF1 knockdown on HU-induced T172-phosphorylation of AMPKα and S345-

phosphorylation of Chk1 in MCF10A cells treated with HU (4 mM, 4 h) or H_2O. Source data are provided as a Source Data file.

Supplementary Figure 3 (related to Figure 3)

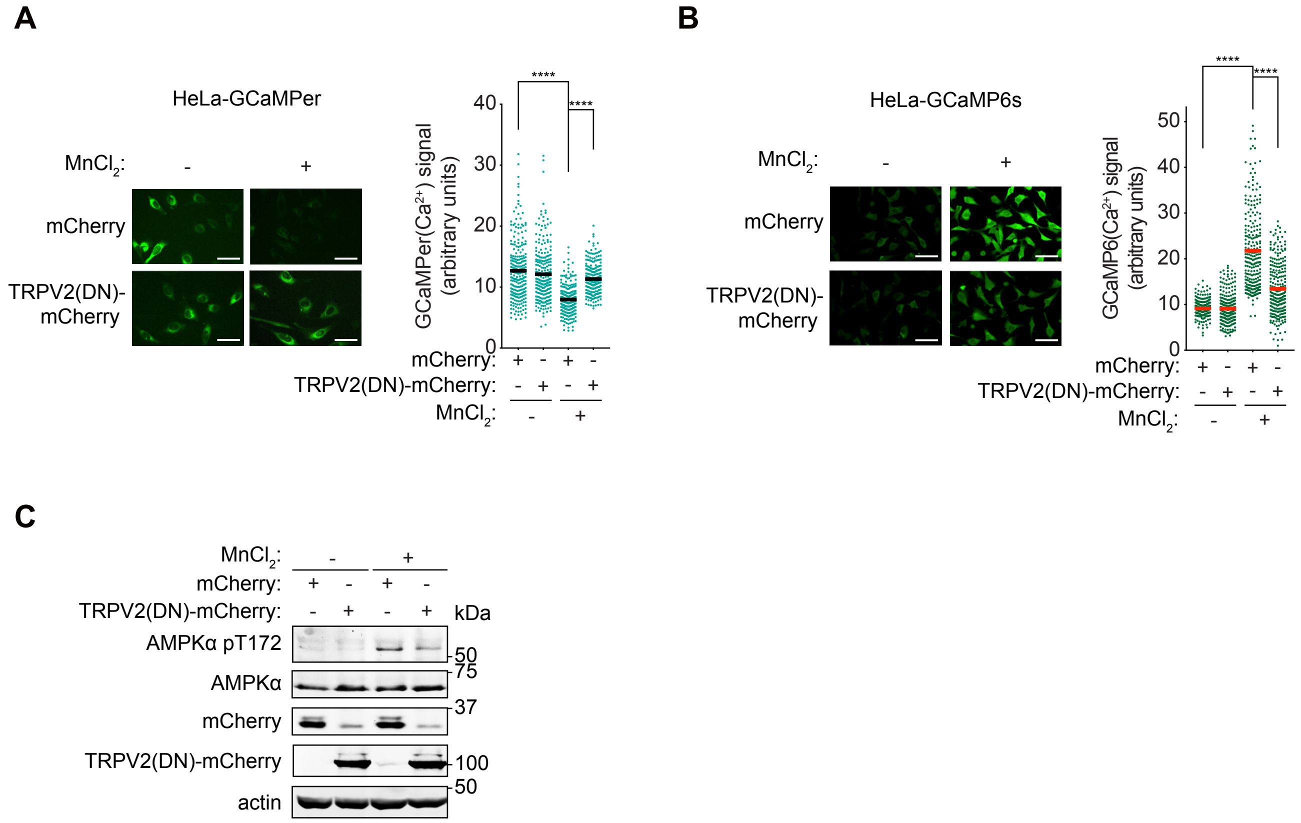

Supplementary Fig.3. TCAF1 is required for Ca^{2+} release in response to cytosolic DNA or direct cGAS activation (Related to Figure 3).

(A) Effects of TRPV2(DN) expression on ER Ca^{2+} release induced by $MnCl_2$. Left panel: Representative images of the GCaMPer signal (scale bar, 25 μm). Right panel: Quantified GcaMPer signal in HeLa cells after $MnCl_2$ treatment (0.5 mM, 1.5 h). 250 cells were scored for each sample. Red bars represent the mean. N=3, ****, p≤0.0001 (two-tailed, unpaired t-test).

(B) Effects of TRPV2(DN) expression on iCa^{2+} elevation induced by $MnCl_2$. Left panel: Representative images of the GcaMP6s signal (scale bar, 25 μm). Right panel: Quantified GcaMP6s signal in HeLa cells after $MnCl_2$ treatment (0.5 mM, 1.5 h). 250 cells were scored for each sample. Red bars represent the mean. N=3, ****, p≤0.0001 (two-tailed, unpaired t-test).

(C) Effect of TRPV2(DN) expression on AMPK T172-phosphorylation induced by $MnCl_2$ treatment (2.5 mM, 4h). Source data are provided as a Source Data file.

Supplementary Figure 4 (related to Figure 5)

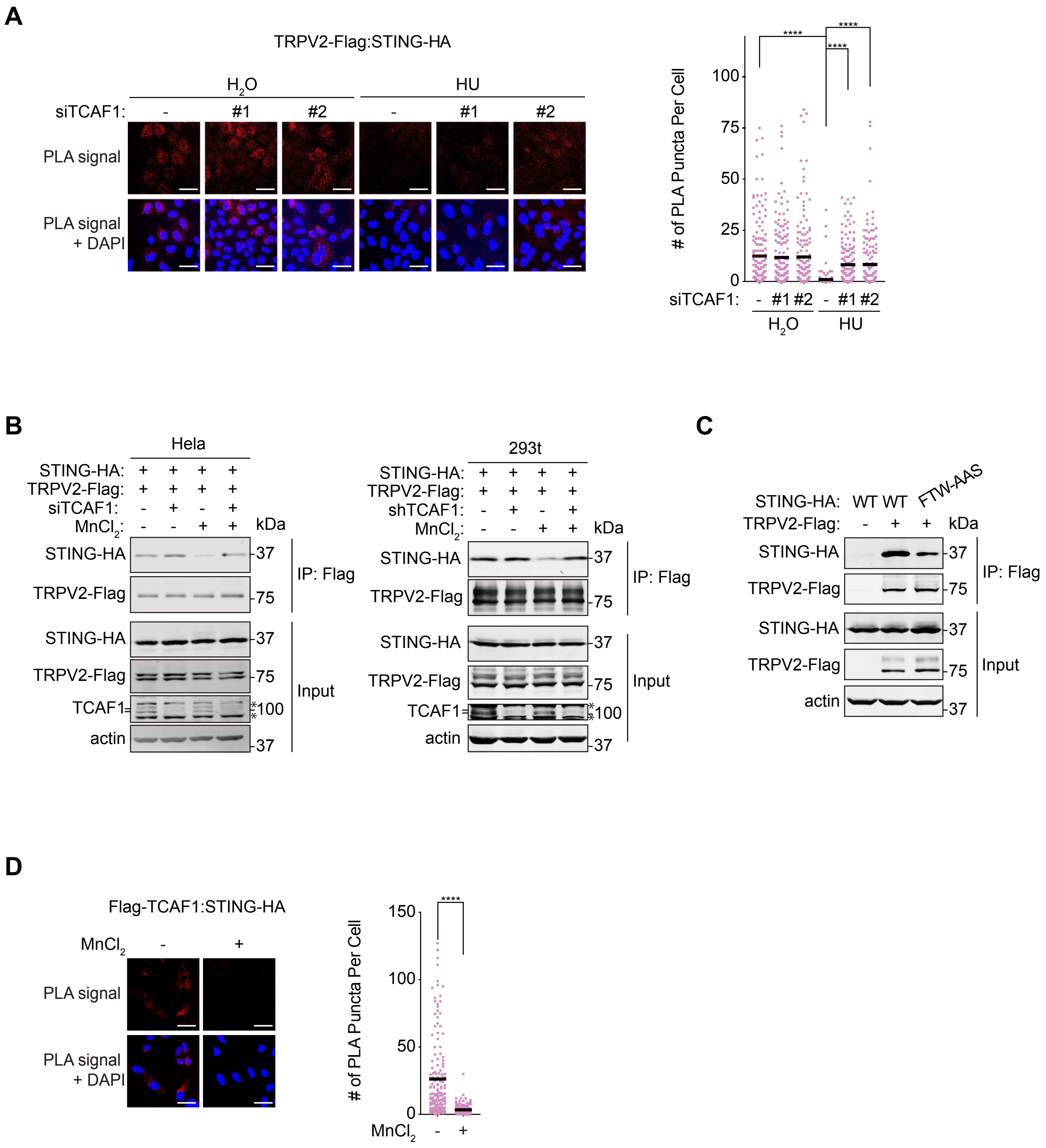

Supplementary Fig.4. TCAF1 promotes STING-TRPV2 dissociation (Related to Figure 5).

(A) Left panel: Representative images of PLA signal for TRPV2-Flag and STING-HA in control-knockdown and TCAF1-knockdown HeLa cells treated with HU (4 mM, 16 h) or H_2O (scale bar, 25 μm). Right panel: Quantified PLA signal of 150 cells in the samples depicted in the left panel. Black bars represent the mean. n=3, ****, p≤0.0001 (two-tailed, unpaired t-test).

(B) Effects of siRNA-mediated (left panel) and shRNA-mediated (right panel) TCAF1 knockdown on the dissociation of STING from TRPV2 induced by $MnCl_2$ (2.5 mM, 4 h) treatment.

(C) W estern blot analysis of co-IP to detect the association of TRPV2-Flag with STING(WT)-HA or with STING(FTW/AAS)-HA.

(D) Effects of $MnCl_2$ treatment on the association between Flag-TCAF1 and STING-HA detected by PLA. Left panel: Representative images of PLA signal of Flag-TCAF1 and STING-HA in HeLa cells treated with $MnCl_2$ (0.5 mM, 1.5 h) (scale bar, 25 μm). Right panel: Quantified PLA signal of 150 cells of the samples depicted in the left panel. Black bars represent the mean. n=3, ****, $p \leqslant 0.0001$ (two-tailed, unpaired t-test).

Note: source data are provided as a Source Data file.
