# Supplementary material for: TCAF1 promotes TRPV2-mediated Ca2+ release in response to cytosolic DNA to protect stressed replication forks
Source: Nat Commun. 2024 May 30;15:4609. doi: 10.1038/s41467-024-48988-6 (PMC11139906; doi:10.1038/s41467-024-48988-6)
Supplement: Supplementary file 3 — Description of Additional Supplementary Files [file 41467_2024_48988_MOESM3_ESM.pdf]

Description of Additional Supplementary Files

File Name: Supplementary Data 1
Description: Original genome-wide CRISPR/Cas9 screen result

File Name: Supplementary Data 2
Description: MAGeCK ranking of genes based on sgRNA depletion after HU treatment in WT HeLa cells

File Name: Supplementary Data 3
Description: Key resources table, including antibodies, chemicals, reagents, kits, oligonucleotide sequences, plasmids and software used in this study.
