# Supplementary material for: TCAF1 promotes TRPV2-mediated Ca2+ release in response to cytosolic DNA to protect stressed replication forks
Source: Nat Commun. 2024 May 30;15:4609. doi: 10.1038/s41467-024-48988-6 (PMC11139906; doi:10.1038/s41467-024-48988-6)
Supplement: Supplementary file 6 — Supplementary Data 3 [file 41467_2024_48988_MOESM6_ESM.docx]

**Supplementary Data 3. Key reagents and resources**

| **REAGENT or RESOURCE** | **SOURCE** | **IDENTIFIER** |
| --- | --- | --- |
| **Antibodies** | | |
| Rabbit polyclonal anti-phospho Exo1 (Ser746) | EMD Millipore | Cat# ABE1066 |
| Mouse mAb anti-Chk1 | Santa Cruz | Cat# sc-8408; RRID: AB_627257 |
| Rabbit mAb anti-Phospho-Chk1 (Ser345) (133D3) | Cell Signaling Technology | Cat# 2348; RRID: AB_331212 |
| Rabbit polyclonal anti-cGAS | Cell Signaling Technology | Cat#15102; RRID: AB_2732795 |
| Rabbit mAb anti-Phospho-AMPKα (Thr172) (40H9) | Cell Signaling Technology | Cat#2535; RRID: AB_331250 |
| Rabbit polyclonal anti-TRPV2 | Sigma-Aldrich | Cat# HPA044993; RRID: AB_10960889 |
| Rabbit mAb anti-HA (C29F4) | Cell Signaling Technology | Cat#3724; RRID: AB_1549585 |
| Mouse mAb anti-FLAG M2 | Cell Signaling Technology | Cat#8146; RRID: AB_10950495 |
| Mouse mAb anti-β-Actin (8H10D10) | Cell Signaling Technology | Cat#3700; RRID: AB_2242334 |
| Rabbit polyclonal anti-β-Tubulin | Abcam | Cat# ab4074; RRID: AB_2288001 |
| Mouse mAb anti-BrdU | BD Pharmingen | Cat# 555627; RRID: AB_10015222 |
| Rat mAb anti-BrdU [BU1/75 (ICR1)] | Abcam | Cat# ab6326; RRID: AB_305426 |
| Rabbit polyclonal anti-DNA2 | Novusbio | Cat# NBP3-12942 |
| Goat anti-Mouse IgG (H+L) Secondary Antibody, DyLight 800 | ThermoFisher | Cat# SA5-10176; RRID: AB_2556756 |
| Goat anti-Rabbit IgG (H+L) Secondary Antibody, DyLight 680 | ThermoFisher | Cat# 35568; RRID: AB_614946 |
| Goat anti-Rabbit IgG (H+L) Secondary Antibody, Alexa Fluor 488 | ThermoFisher | Cat# A-11008; RRID: AB_143165 |
| Goat anti-Mouse IgG (H+L) Secondary Antibody, Alexa Fluor 488 | ThermoFisher | Cat# A-11001; RRID: AB_2534069 |
|  |  |  |
| **Chemicals, reagents and kits** | | |
| Hydroxyurea | Sigma-Aldrich | Cat#: H8627, CAS: 127-07-1 |
| Bleomycin | Sigma-Aldrich | Cat#: 15361, CAS: 9041-93-4 |
| Camptothecin (CPT) | MedChemExpress | Cat#: M9948, CAS: 299953-00-7 |
| Mirin | Sigma-Aldrich | Cat#: M3634, CAS: 13446-34-9 |
| Manganese (II) chloride tetrahydrate | Sigma-Aldrich | Cat#: M3634, CAS: 13446-34-9 |
| Nocodazole | Sigma-Aldrich | Cat#: M1404, CAS: 31430-18-9 |
| Thymidine | EDM Millipore | Cat#: AC226740050, CAS: 50-89-5 |
| 2’3’-cGAMP | Cell Signaling Technology | Cat#: 35573, CAS: 1441190-66-4 |
| 5-Iodo-2′-deoxyuridine (IdU) | Sigma-Aldrich | Cat#: I7125, CAS: 54-42-2 |
| 5-Chloro-2′-deoxyuridine (CldU) | Sigma-Aldrich | Cat#: C6891, CAS: 50-90-8 |
| 5-BrdU | ThermoFisher | Cat#: B9285, CAS: 59-14-3 |
| Hoechst 33342 | ThermoFisher | Cat#: H3570, CAS: 875756-97-1 |
| DAPI | Sigma-Aldrich | Cat#: D9542, CAS: 28718-90-3 |
| Crystal Violet | Sigma-Aldrich | Cat#: C0775, CAS: 548-62-9 |
| Protease/phosphatase inhibitor | Cell Signaling Technology | Cat#: 5872 |
| Anti-FLAG® M2 Magnetic Beads | Sigma-Aldrich | Cat#: M8823 |
| Lipofectamine™ RNAiMAX Transfection Reagent | ThermoFisher | Cat#: 13778100 |
| TransIT®-LT1 Transfection Reagent | Mirus | Cat#: MIR2300 |
| 2’3’-cGAMP ELISA Kit | Cayman Chemical | Cat#: 501700 |
| Duolink In Situ Detection Reagents Red | Sigma-Aldrich | Cat#: DUO92008 |
| Duolink In Situ PLA Probe Anti-Mouse Minus | Sigma-Aldrich | Cat#: DUO92004 |
| Duolink In Situ PLA Probe Anti-Rabbit Plus | Sigma-Aldrich | Cat#: DUO92002 |
|  |  |  |
| **Cell lines** | | |
| HeLa | ATCC | Cat#: CCL-2 |
| U2OS | ATCC | Cat#: HTB-96 |
| HEK 293T | ATCC | Cat#: CRL-11268 |
| MCF 10A | ATCC | Cat#: CRL-10317 |
|  |  |  |
| **sgRNA library, oligonucleotides** | | |
| GeCKOv2 CRISPR knockout pooled library | Addgene | Cat#: 1000000049 |
| Stealth RNAi Luciferase Reporter Control (Control) | Invitrogen | REF: 12935146 |
| siRNA for Exo1 | Thermo Fisher | CTTTTGAACAGATCGATGA |
| siRNA for TCAF1#1 | Thermo Fisher | GGTTGAGCCAGAAGTGAAA |
| siRNA for TCAF1#2 | Thermo Fisher | GGACCGCACTGGAAACGTA |
| siRNA for cGAS | Thermo Fisher | GGAAGAAATTAACGACATT |
| siRNA for DNA2 | Thermo Fisher | AUAGCCAGUAGUAUUCGAU |
| shRNA for Luciferase | This paper | GAATCGTCGTATGCAGTGAAA |
| shRNA for TCAF1 #1 | This paper | GCATGATAATGAAAGAGTGAA |
| shRNA for TCAF1 #2 | This paper | CCACTATTGATACCAGTTAAA |
| shRNA for STING #1 | This paper | GCCCGGATTCGAACTTACAAT |
| shRNA for STING #2 | This paper | GTCCAGGACTTGACATCTTAA |
| shRNA for TRPV2 | This paper | AGCCGGATCCAAACCGATTTG |
| shRNA for TREX1 #1 | This paper | AACACGGCCCAAGGAAGAGCT |
| shRNA for TREX1 #2 | This paper | AAGACCATCTGCTGTCACAAC |
| qPCR primers for TCAF2 | This paper | Forward: ACCACGAGAATGGGAACTTG  Reverse: GAGCCTGTGCAGGGATATGT |
| qPCR primers for human GAPDH | This paper | Forward: AACAGCGACACCCACTCCTC  Reverse: GGAGGGGAGATTCAGTGTGGT |
| Illumina Sequencing Forward primer 1 | This paper | AATGATACGGCGACCACCGAGATCTACACTCTTTCCCTACACGACGCTCTTCCGATCTtcttgtggaaaggacgaaacaccg |
| Illumina Sequencing Forward primer 2 | This paper | AATGATACGGCGACCACCGAGATCTACACTCTTTCCCTACACGACGCTCTTCCGATCTGtcttgtggaaaggacgaaacaccg |
| Illumina Sequencing Forward primer 3 | This paper | AATGATACGGCGACCACCGAGATCTACACTCTTTCCCTACACGACGCTCTTCCGATCTTAtcttgtggaaaggacgaaacaccg |
| Illumina Sequencing Forward primer 4 | This paper | AATGATACGGCGACCACCGAGATCTACACTCTTTCCCTACACGACGCTCTTCCGATCTCAGtcttgtggaaaggacgaaacaccg |
| Illumina Sequencing Forward primer 5 | This paper | AATGATACGGCGACCACCGAGATCTACACTCTTTCCCTACACGACGCTCTTCCGATCTAGAGtcttgtggaaaggacgaaacaccg |
| Illumina Sequencing Reverse Index primer 1 | This paper | CAAGCAGAAGACGGCATACGAGATAACCTCAGTGACTGGAGTTCAGACGTGTGCTCTTCCGATCTtctactattctttcccctgcactgt |
| Illumina Sequencing Reverse Index primer 2 | This paper | CAAGCAGAAGACGGCATACGAGATTCTAAGCGTGACTGGAGTTCAGACGTGTGCTCTTCCGATCTtctactattctttcccctgcactgt |
| Illumina Sequencing Reverse Index primer 3 | This paper | CAAGCAGAAGACGGCATACGAGATCTGTCATGTGACTGGAGTTCAGACGTGTGCTCTTCCGATCTtctactattctttcccctgcactgt |
| Illumina Sequencing Reverse Index primer 4 | This paper | CAAGCAGAAGACGGCATACGAGATGGAGGTGGTGACTGGAGTTCAGACGTGTGCTCTTCCGATCTtctactattctttcccctgcactgt |
| sgRNA for TCAF1#1 | This paper | CACTCACCCAGCTTGTAGTA |
| sgRNA for TCAF1#2 | This paper | GCACCACAACCTTGCCTCTG |
| sgRNA for TCAF1#3 | This paper | CCTTACCTTCAGGTCGGCAG |
| sgRNA for Exo1 | This paper | GGAATATTGCTCTTTGAACA |
|  |  |  |
| **Plasmids** | | |
| Lenti_CMV-Cas9-P2A-HygR | Addgene | Cat#: 52962; RRID: Addgene_164133 |
| LentiCas9-Blast | Addgene | Cat#: 52962; RRID: Addgene_52962 |
| Human CRISPR Knockout Pooled Library (GeCKO v2) | Addgene | Cat#: (Pooled Library #1000000048, #1000000049); RRID: Addgene_1000000048, Addgene_1000000049 |
| psPAX2 | Addgene | Cat#: 12260; RRID: Addgene_12260 |
| pMD2.G | Addgene | Cat#: 12259; RRID: Addgene_12259 |
| pMOS003-lenti-CMV-GCaMPer | Addgene | Cat#: 65227; RRID: Addgene_65227 |
| pBOB-GCaMP6s | This paper | N/A |
| pCDH-TRPV2-mCherry (wild-type or mutants) | PMID: 36696898 | N/A |
| pCDH-Flag-TCAF1 (wild-type or mutants) | This paper | N/A |
| pCDH-3XFlag-TCAF1 (wild-type or mutants) | This paper | N/A |
| pCDH-3XFlag-TCAF1-HA | This paper | N/A |
| pCDH-TRPV2-Flag | This paper | N/A |
| pCDH-STING-HA | This paper | N/A |
| pLKO.1-shRNAs | This paper | N/A |
| pGEX-4T-2-TCAF1 (1-279)aa | This paper | N/A |
|  |  |  |
| **Software and algorithms** | | |
| GraphPad Prism | GraphPad | https://www.graphpad.com/ |
| Fiji-ImageJ | ImageJ | https://imagej.nih.gov/ij/ |
| FlowJo | FlowJo | https://www.flowjo.com/ |
| Image Studio™ Lite | LI-COR Biosciences | https://www.licor.com/bio/products/software/image_studio_lite/ |
