# Supplementary material for: TCAF1 promotes TRPV2-mediated Ca2+ release in response to cytosolic DNA to protect stressed replication forks
Source: Nat Commun. 2024 May 30;15:4609. doi: 10.1038/s41467-024-48988-6 (PMC11139906; doi:10.1038/s41467-024-48988-6)
Supplement: Supplementary file 7 — Reporting Summary [file 41467_2024_48988_MOESM7_ESM.pdf]

nature portfolio

Corresponding author(s): Zhongsheng You
Last updated by author(s): May 14, 2024

# Reporting Summary

Nature Portfolio wishes to improve the reproducibility of the work that we publish. This form provides structure for consistency and transparency in reporting. For further information on Nature Portfolio policies, see our Editorial Policies and the Editorial Policy Checklist.

## Statistics

For all statistical analyses, confirm that the following items are present in the figure legend, table legend, main text, or Methods section.

| n/a | Confirmed | |
|---|---|---|
| ☐ | ☒ | The exact sample size (*n*) for each experimental group/condition, given as a discrete number and unit of measurement |
| ☐ | ☒ | A statement on whether measurements were taken from distinct samples or whether the same sample was measured repeatedly |
| ☐ | ☒ | The statistical test(s) used AND whether they are one- or two-sided<br>*Only common tests should be described solely by name; describe more complex techniques in the Methods section.* |
| ☐ | ☒ | A description of all covariates tested |
| ☒ | ☐ | A description of any assumptions or corrections, such as tests of normality and adjustment for multiple comparisons |
| ☐ | ☒ | A full description of the statistical parameters including central tendency (e.g. means) or other basic estimates (e.g. regression coefficient) AND variation (e.g. standard deviation) or associated estimates of uncertainty (e.g. confidence intervals) |
| ☒ | ☐ | For null hypothesis testing, the test statistic (e.g. *F*, *t*, *r*) with confidence intervals, effect sizes, degrees of freedom and *P* value noted<br>*Give P values as exact values whenever suitable.* |
| ☒ | ☐ | For Bayesian analysis, information on the choice of priors and Markov chain Monte Carlo settings |
| ☒ | ☐ | For hierarchical and complex designs, identification of the appropriate level for tests and full reporting of outcomes |
| ☒ | ☐ | Estimates of effect sizes (e.g. Cohen's *d*, Pearson's *r*), indicating how they were calculated |

*Our web collection on statistics for biologists contains articles on many of the points above.*

## Software and code

Policy information about availability of computer code

| | |
|---|---|
| Data collection | MetaMorph software was used to acquire widefield images.<br>Gen5 was used to acquire OD450 value for ELISA. |
| Data analysis | ImageJ software was used to analyze pseudocolor images.<br>Western blot images were analyzed with Image Studio Lite.<br>Flow cytometry data was analyzed with FlowJo.<br>GraphPad Prism was used to do statisical analysis and create graphs. |

For manuscripts utilizing custom algorithms or software that are central to the research but not yet described in published literature, software must be made available to editors and reviewers. We strongly encourage code deposition in a community repository (e.g. GitHub). See the Nature Portfolio guidelines for submitting code & software for further information.

## Data

Policy information about availability of data

All manuscripts must include a data availability statement. This statement should provide the following information, where applicable:

- Accession codes, unique identifiers, or web links for publicly available datasets
- A description of any restrictions on data availability
- For clinical datasets or third party data, please ensure that the statement adheres to our policy

All data supporting the described findings are available in this paper and in the Supplementary Information/Source Data file. The source data, including original Western blot and microscopic signal quantifications are provided in the Source Data file. The raw Genome-wide CRISPR/Cas9 screen sequencing data generated in this study have been deposited in the Gene Expression Omnibus (GEO) database with the accession code GSE244205. The processed sequencing data are provided in the Supplementary Data 1 and 2 files.

## Research involving human participants, their data, or biological material

Policy information about studies with human participants or human data. See also policy information about sex, gender (identity/presentation), and sexual orientation and race, ethnicity and racism.

| | |
|---|---|
| Reporting on sex and gender | No human participants in this study. |
| Reporting on race, ethnicity, or other socially relevant groupings | No human participants in this study. |
| Population characteristics | No human participants in this study. |
| Recruitment | No human participants in this study. |
| Ethics oversight | No human participants in this study. |

Note that full information on the approval of the study protocol must also be provided in the manuscript.

# Field-specific reporting

Please select the one below that is the best fit for your research. If you are not sure, read the appropriate sections before making your selection.

☒ Life sciences ☐ Behavioural & social sciences ☐ Ecological, evolutionary & environmental sciences

For a reference copy of the document with all sections, see nature.com/documents/nr-reporting-summary-flat.pdf

# Life sciences study design

All studies must disclose on these points even when the disclosure is negative.

| | |
|---|---|
| Sample size | Sample size was not pre-determined.<br>For imaging analysis of nondenaturing BrdU IF staining to detect ssDNA formation, 1000 cells per sample were analyzed in each experiment.<br>For DNA fiber assay to measuring fork resection, on average 150 tracts per sample were analyzed.<br>For Metaphase chromosome spreading assay, 150 Metaphase cells per sample were analyzed.<br>For live cell GCaMP6s and GCaMPer imaging, 250 cells per sample were analyzed.<br>For proximity ligation assay, 150 cells per sample were analyzed.<br>Sample size and number are clearly stated in the figures legend or in the Methods section.<br>The sample sizes used were based on our published research and that of others. |
| Data exclusions | Outlier analysis was performed for Figures lC and 4B. No data were excluded from the analysis in other figures. |
| Replication | All experiments were performed three or more times independently under similar conditions, except experiments shown in Fig lA (CRISPR screen), which was performed once and Fig 4A (cGAMP ELISA), which was performed twice. |
| Randomization | Samples were randomly allocated to different groups. |
| Blinding | Blinded quantification was performed by multiple investigators when samples exhibited less obvious signal differences. |

# Reporting for specific materials, systems and methods

We require information from authors about some types of materials, experimental systems and methods used in many studies. Here, indicate whether each material, system or method listed is relevant to your study. If you are not sure if a list item applies to your research, read the appropriate section before selecting a response.

## Materials & experimental systems

| n/a | Involved in the study |
|---|---|
| ☐ | ☒ Antibodies |
| ☐ | ☒ Eukaryotic cell lines |
| ☒ | ☐ Palaeontology and archaeology |
| ☒ | ☐ Animals and other organisms |
| ☒ | ☐ Clinical data |
| ☒ | ☐ Dual use research of concern |
| ☒ | ☐ Plants |

## Methods

| n/a | Involved in the study |
|---|---|
| ☒ | ☐ ChIP-seq |
| ☐ | ☒ Flow cytometry |
| ☒ | ☐ MRI-based neuroimaging |

# Antibodies

Antibodies used

Full information about the antibodies used in this study is provided in Materials and Methods section.
Antibodies
Rabbit polyclonal anti-phospho Exo1 (Ser746)
EMD Millipore
Cat# ABE1066
Mouse mAb anti-Chk1
Santa Cruz
Cat# sc-8408; RRID: AB_627257
Rabbit mAb anti-Phospho-Chk1 (Ser345) (133D3)
Cell Signaling Technology
Cat# 2348; RRID: AB_331212
Rabbit polyclonal anti-cGAS
Cell Signaling Technology
Cat#15102; RRID: AB_2732795
Rabbit mAb anti-Phospho-AMPKα (Thr172) (40H9)
Cell Signaling Technology
Cat#2535; RRID: AB_331250
Rabbit polyclonal anti-TRPV2
Sigma-Aldrich
Cat# HPA044993; RRID: AB_10960889
Rabbit mAb anti-HA (C29F4)
Cell Signaling Technology
Cat#3724; RRID: AB_1549585
Mouse mAb anti-FLAG M2
Cell Signaling Technology
Cat#8146; RRID: AB_10950495
Mouse mAb anti-β-Actin (8H10D10)
Cell Signaling Technology
Cat#3700; RRID: AB_2242334
Rabbit polyclonal anti-β-Tubulin
Abcam
Cat# ab4074; RRID: AB_2288001
Mouse mAb anti-BrdU
BD Pharmingen
Cat# 555627; RRID: AB_10015222
Rat mAb anti-BrdU [BU1/75 (ICR1)]
Abcam
Cat# ab6326; RRID: AB_305426
Rabbit polyclonal anti-DNA2
Novusbio
Cat# NBP3-12942
Goat anti-Mouse IgG (H+L) Secondary Antibody, DyLight 800
ThermoFisher
Cat# SA5-10176; RRID: AB_2556756
Goat anti-Rabbit IgG (H+L) Secondary Antibody, DyLight 680
ThermoFisher
Cat# 35568; RRID: AB_614946
Goat anti-Rabbit IgG (H+L) Secondary Antibody, Alexa Fluor 488
ThermoFisher
Cat# A-11008; RRID: AB_143165
Goat anti-Mouse IgG (H+L) Secondary Antibody, Alexa Fluor 488
ThermoFisher
Cat# A-11001; RRID: AB_2534069

| | |
|---|---|
| Validation | Full information about the antibodies used in this study is provided in Materials and Methods section. Please see our answer in the form. |

## Eukaryotic cell lines

Policy information about cell lines and Sex and Gender in Research

| | |
|---|---|
| Cell line source(s) | Full information about the cell lines used in this study is provided in Materials and Methods section.<br>HeLa<br>ATCC<br>Cat#: CCL-2<br>U2OS<br>ATCC<br>Cat#: HTB-96<br>HEK 293T<br>ATCC<br>Cat#: CRL-11268<br>MCF 10A<br>ATCC<br>Cat#: CRL-10317 |
| Authentication | Cell lines were authenticated using the short tandem repeat genotyping method |
| Mycoplasma contamination | Confirm negative |
| Commonly misidentified lines<br>(See ICLAC register) | HeLa. HeLa cells are commonly used for replication stress studies. Like normal cells, this cell line has all the components of the components, including TCAF1, cGAS, STING and TRPV2, in the Ca2+ pathway. U2OS, 293T and MCF10A cells are not commonly misidentified cell lines. |

## Flow Cytometry

### Plots

Confirm that:

☒ The axis labels state the marker and fluorochrome used (e.g. CD4-FITC).

☒ The axis scales are clearly visible. Include numbers along axes only for bottom left plot of group (a 'group' is an analysis of identical markers).

☐ All plots are contour plots with outliers or pseudocolor plots.

☐ A numerical value for number of cells or percentage (with statistics) is provided.

### Methodology

| | |
|---|---|
| Sample preparation | For the cell cycle analysis in Figure S1E, cells were pulsed with BrdU (20 μM) for 30 min, and then trypsinized. After wash with PBS, cells were fixed in 70% ethanol at −20°C overnight. Subsequently, cells were pelleted down and then incubated with 2 N HCl/0.5% Triton X-100 for 30 min at room temperature to denature DNA followed by neutralization in 0.1 M sodium tetraborate (pH 8.5). Cells were next incubated with mouse anti-BrdU antibody (1:400, BD Biosciences, 347580) in antibody dilution buffer (PBS + 0.5% Tween 20 + 1% BSA) overnight at 4°C. After incubation, cells were washed 3 times with PBS containing 1% BSA and then incubated with Alexa Fluor 488-conjugated goat anti-mouse IgG (1:500, Thermofisher, A-11001) for 1 h. After wash with PBS containing 1% BSA, cells were resuspended in PBS containing propidium iodide (20 μg/ml) and RNase A (200 μg/ml) and incubated at 37°C for 30 min in the dark. |
| Instrument | BD FACScan5 Flow Cytometer |
| Software | FlowJo software |
| Cell population abundance | not applicable |
| Gating strategy | Cell Cycle Analysis by Propidium Iodide (PI)staining: 1. Exclusion of cell debris by forward scatter (FSC) and side scatter (SSC); 2. Exclusion of clumps and doublets by forward scatter width (FSC-W) and height (FSC-H); |

☐ Tick this box to confirm that a figure exemplifying the gating strategy is provided in the Supplementary Information.
